# Expression of BVDV E2 protein in CHO-S cells and development of an indirect ELISA for serological detection

**DOI:** 10.3389/fcimb.2025.1631027

**Published:** 2025-07-24

**Authors:** Jiahui Wang, Ao Zhang, Zhiwei Hou, Bin Tan, Shuqin Zhang

**Affiliations:** Institute of Special Animal and Plant Sciences, Chinese Academy of Agricultural Sciences, Changchun, China

**Keywords:** bovine viral diarrhea virus (BVDV), E2 protein, indirect ELISA, serological diagnosis, recombinant antigen

## Abstract

Bovine viral diarrhea virus (BVDV) causes ongoing economic losses to the livestock industry. Monitoring antibodies via enzyme-linked immunosorbent assay (ELISA) is a key tool for ensuring the eradication of BVDV from cattle herds. We developed an indirect ELISA (rE2-iELISA) using CHO-S-expressed recombinant E2 protein, the major immunogenic glycoprotein mediating viral attachment and immune evasion. Optimized assay conditions included: 0.4 μg/well antigen coating, 5% BSA blocking, 1:100 serum dilution, and 1:5000 secondary antibody dilution. The assay demonstrated exclusive specificity for BVDV-1 and BVDV-2 with detection sensitivity to 1:1,500 serum dilution. Validation revealed exceptional diagnostic performance: ROC analysis showed 0.998 AUC (cutoff=0.125), 94.8% concordance with IDEXX ELISA, and the intra- and inter-batch coefficient of variation are both less than 5%. The experimental results indicate that the indirect ELISA detection method based on BVDV rE2 exhibits good sensitivity, specificity, and stability. And a stable serological tool for BVDV surveillance and vaccine efficacy evaluation in cattle populations.

## Background

Bovine viral diarrhea virus (BVDV) is a single RNA virus of the genus Pestivirus in the family Flavivirus. Under transmission electron microscopy, BVDV particles are circular or elliptical in structure, with a diameter of approximately 50 nm (Ambinder et al., 1986). There are two biotypes of BVDV: cellular (CP) and non-cellular (NCP).BVDV can infect cattle during early embryonic development, resulting in persistently infected (PI) animals (Baker, 1995).

BVDV is categorized into three different genotypes: BVDV-1, BVDV-2, and the recently discovered BVDV-3, which is mainly found in regions such as Brazil and Southeast Asia (Elisa Riitho et al., 2020). The viral genome contains an open reading frame (ORF) that encodes four structural proteins, namely C, E1, E2, and E3, and eight nonstructural proteins, including Npro, p7, NS2, NS3, NS4A, NS4B, NS5A, and NS5B. This ORF is located between the 5’-UTR and 3’-UTR regions (Yu et al., 2000; Becher et al., 2000; Makoschey et al., 2004). As an important component of the viral envelope, E2 is an antigenic protein of BVDV, which mainly produces neutralizing antibodies against the virus and is a molecular target for BVDV genotyping and diagnosis (Henningson et al., 2013), and is also a receptor-binding protein of BVDV, whose βG hairpin structural domain plays a key role in the binding process of the virus and the receptor. The BVDV E2 protein is critical for the BVDV antigenic properties and plays a key role in immune neutralization.

Mammalian expression systems play a crucial role in the discovery of new genes, the study of protein structure and function, and the production of drugs and vaccines (Kamachi and Omasa, 2018).CHO cellular expression system is one of the most representative mammalian cellular expression systems currently available. CHO fine does not secrete, or rarely secretes, endogenous proteins, which contributes to the purification of recombinant proteins. CHO cells have been extensively studied over the past 20 years and have been a safe host for the expression of exogenous proteins. Therefore, they are more likely to receive regulatory approval from agencies such as the U.S. Food and Drug Administration (FDA) for the production of therapeutic proteins (Zhu et al., 2012).

Bovine viral diarrhea virus (BVDV) is widely prevalent in China, with high infection rates and a lack of effective treatments (Ran et al., 2019). In many countries, BVDV control strategies are built around identifying PI animals, and early detection, especially after birth, is essential for successful BVDV management. According to data from the Chinese National Veterinary Drug Database, as of May 2025, three inactivated vaccines have been approved for registration, including a monovalent vaccine made from the BVDV NM01 strain (type 1), a bivalent vaccine made from the BVDV NMG strain and the IBRV LY strain, and a bivalent vaccine made from the BVDV NM01 strain and the IBRV LN01/08 strain. Vaccination is one of the primary strategies for prevention, helping to control and eradicate many viral diseases, including BVDV. He, Y (He et al., 2023) conducted longitudinal surveillance of BVDV-neutralizing antibody titers in vaccinated cattle populations across selected Chinese provinces and municipalities from 2018 to 2021. The results demonstrated a consistent year-on-year increase in BVDV-neutralizing antibody levels, indicating that vaccination significantly enhanced the overall immune status of the cattle herds. Liu, Y (Liu, 2023) found in his research that the low detection rate of BVDV in Hebei, China, may be related to the high level of attention paid to BVDV in the cattle industry in recent years and the control measures taken by large-scale cattle farms, such as the timely removal of PI cows and vaccination. A range of diagnostic methods is currently available, such as virus isolation, immunohistochemistry, antigen-capture ELISA, and RT-PCR, each of which has its unique strengths and limitations and is suitable for different diagnostic settings. ELISA is favored because of its simplicity of procedure, high sensitivity, and specificity, making it a popular choice for the detection of BVDV in cattle. In this study, we used the CHO-S eukaryotic expression system to produce recombinant E2 (rE2) protein for BVDV. We developed an indirect ELISA method using the rE2 protein as an encapsulated antigen to improve the accuracy of BVDV antibody detection. We provide technical support for clinical diagnosis and evaluation of immunization effects and other related studies.

## Materials and methods

### Virus strain, plasmids, and antibodies

BVDV JL-1 strain (GenBank accession number: KF501393.1) is stored in our laboratory. The pcDNA3.1 vector was purchased from Takara Biomedical Technology Co., Ltd. (Beijing, China), His-Tag rabbit polyclonal antibody (Epizyme, Shanghai, China), goat anti-rabbit IgG (H+L), and HRP-labeled goat anti-bovine IgG (Sangon Biotech, Shanghai, China). His-Tag rabbit polyclonal antibody (Epizyme, Shanghai, China), goat anti-rabbit IgG (H+L), and HRP-labeled goat anti-bovine IgG (Sangon Biotech, Shanghai, China). Mouse anti-BVDV E2 specific monoclonal antibody (VMRD, Pullman, Washington, USA). BVDV Ab Test (IDEXX Laboratories, Inc., Westbrook, ME, USA). The iELISA detection method was established using positive and negative standard sera prepared by this laboratory. Classical swine fever virus (CSFV), bovine viral diarrhea virus (BVDV-1 and BVDV-2), bovine paramyxovirus type 3 (BPIV-3), and infectious bovine rhinotracheitis virus (IBRV) were all prepared and stored by this laboratory.

### Construction of the recombinant pcDNA3.1−E2 plasmid vector

The full-length gene sequence of the E2 protein was amplified from the genomic RNA of the BVDV JL-1 strain (GenBank accession no KF501393.1) using high-fidelity reverse transcription-polymerase chain reaction (RT-PCR). The linearized pcDNA3.1(+) vector and the amplified gene fragment were ligated using a seamless cloning strategy, and the resulting construct was transformed into chemoreceptor DH5α cells. To enhance the translational efficiency and secretory expression of the target protein, the Kozak sequence (GCCACC) and the human CD5 signal peptide sequence were inserted before the start codon. In addition, a precisely designed 6× His tag (CATCATCATCATCATCAT) was introduced at the C-terminus to facilitate immunological detection and nickel column affinity purification of recombinant proteins. The constructed plasmids were extracted, verified by 1% agarose gel electrophoresis, and sequenced (Jilin Kumei Co., Ltd., Changchun, China). The target genes and sequences obtained were analyzed through multiple sequence comparisons using SnapGene software (version 6.0.2). The plasmid with 100% sequence identity was named pcDNA3.1-E2 and stored in glycerol at -80°C protected from light for later use.

### Expression and purification of recombinant proteins

Transient transfection for expression of rE2 was performed using the ExpiCHO™ Expression System (Thermo Fisher Scientific, USA). The cell supernatant was collected after transfection, and the cellular supernatant was analyzed using a pre-equilibrated Ni-Sepharose HisTrap™ excel column (5 mL) through an ÄKTA explorer 100 chromatography system (GE Healthcare, USA) in a 20 mM PBS (pH 7.4) buffer system. rE2 was purified from the purified protein samples by mixing with 2× reduced SDS sample buffer and heating for 10 min at 95°C in a metal bath to denature the protein fully. Proteins were separated by 10% Tris-Glycine SDS-PAGE, followed by staining with 0.25% Coomassie Brilliant Blue R-250 for 1 h. For protein blotting analysis, proteins resolved electrophoretically in SDS-PAGE gels were electro-transferred onto PVDF membranes (Merck Millipore, USA) at a constant voltage of 15 V and stained at room temperature with 5% skim milk-TBST blocking buffer (EpiZyme, China) for 60 min. The PVDF membranes were incubated with a 1:5,000 dilution of His-tag rabbit polyclonal antibody (EpiZyme, China) at 4°C overnight. The membranes were then incubated with HRP-coupled goat anti-rabbit IgG (EpiZyme, China) at a dilution of 1:8,000 for 1 h at room temperature, and the bands were visualized by chemiluminescence using ultrasensitive ECL reagent (Beyotime, China). For glycosylation analysis, 10 µg of rE2 protein was used, and the protein was denatured with 1× glycoprotein denaturation buffer (containing 0.5% SDS and 40 mM DTT) at 100°C for 10 min. After the addition of NP-40 at a final concentration of 1% and 1× GlycoBuffer 2, 500 U PNGase F (New England BioLabs, USA) was added, and the reaction mixture was incubated for 1 h at 37°C. The previously described Western Blot process validated the reaction products.

### Development and optimization of the rE2−based iELISA

To establish the iELISA method, a checkerboard titration experiment was first conducted to determine the optimal working conditions for the coated antigen and serum samples, specifically including: purifying rE2 protein was serially diluted to 8, 7, 6, 5, and 4 µg/mL as the coating concentration; BVDV-positive control serum was diluted in a 1:40 to 1:140 ratio; HRP-labeled goat anti-bovine IgG (Sangon Biotech, China) was diluted in a gradient from 1:5000 to 1:9000; simultaneously, the effects of seven blocking agents (5% HS, 3% HS, 5% BSA, 3% BSA, 5% skim milk powder, 5% gelatin, and 3% gelatin) and secondary antibody incubation time (40, 60, 90, and 120 minutes) on assay performance were systematically evaluated. The standard operating procedure is as follows: Coat overnight at 4°C with 100 µL of rE2 antigen (dissolved in 0.05 M carbonate buffer, pH 9.6), wash five times with PBST (containing 0.05% Tween-20), blocked with 200 µL of optimized blocking solution at 37°C for 2 hours; added 100 µL of diluted serum and incubated at 37°C for 3 hours, washed, then added 100 µL of optimized concentration secondary antibody and reacted at 37°C for 1 hour; after TMB color development for 15 minutes, the reaction was terminated with 50 µL of 2 M TMAH, and immediately measure the absorbance at 450 nm (reference wavelength 630 nm) using an enzyme-linked immunosorbent assay (ELISA) reader. All experimental conditions were validated through three independent replicate experiments, and the optimal reaction system was determined using the positive/negative control OD450 ratio (P/N ≥ 2.1) as the criterion.

### rE2 protein-based iELISA and ROC analysis

A rE2-based ELISA was utilized for identifying specific antibodies against BVDV in 160 serum samples, comprising 80 positive and 80 negative bovine serum samples, all of which were confirmed by VNT. For ELISA testing, the samples were diluted using TBST. The cutoff value for the iELISA was determined through receiver operating characteristic (ROC) analysis, and the results were evaluated using GraphPad software. The relative sensitivity and specificity were computed, with the cutoff value established based on the maximum Youden index obtained from the ROC analysis.

### Evaluation of specificity, sensitivity, and technical reproducibility

The developed ELISA method is used to detect positive sera for IBRV, BRSV, BPIV-3, and CSFV, as well as positive and negative sera for BVDV. To assess sensitivity, BVDV-positive sera were diluted from 1:100 to 1:5,000 to determine the highest dilution at which the serum could still be detected. Each sample was tested in duplicate, and the average value was calculated. Specificity and sensitivity were assessed using the same formula as for cutoff value analysis: (sample OD value − negative control OD value)/(positive control OD value − negative control OD value). Intra-assay reproducibility was evaluated by testing the same sample in five different wells on the same microplate. Inter-assay reproducibility was assessed by testing the same set of serum samples across five independently produced ELISA plate batches. The coefficient of variation (CV%) for precision analysis was calculated.

### Testing of clinical samples

Between 2022 and 2024, this laboratory randomly collected 500 serum samples were tested them for BVDV antibodies using three methods: rE2-iELISA, serum neutralization (SN) (**Table 1**), and a commercial IDEXX-ELISA kit. Discordant results were confirmed by Western blot (WB) using BVDV whole lysate as the antigen. Statistical agreement between the methods was assessed using Cohen’s kappa coefficient (κ) and percent concordance.

**Table 1:** Serum samples employed in this study.

Sample source (province)	Location	Breed of cattle	Production Stage Classification	Immunization status (BVDV)	VNT Positive
Jilin	Farm 1	Yanbian Cattle	Calves Young Cattle	Unvaccinated	62/70 (88.6%)
Heilongjiang	Farm 2	Chinese Simmental	Adult Cattle	Partial vaccination	74/82 (90.2%)
	Farm 3	Holstein	Growing Cattle	Partial vaccination	76/82 (92.7%)
Liaoning	Farm 4	Chinese Simmental	Calves Adult Cattle	Unvaccinated	46/66 (69.7%)
	Farm 5	Holstein	Adult Cattle	Partial vaccination	83/97 (85.6%)
	Farm 6	Holstein	Calves Growing Cattle	Partial vaccination	89/103 (86.4%)

## Results

### Expression and purification of recombinant BVDV E2 protein

**Figure 1:**
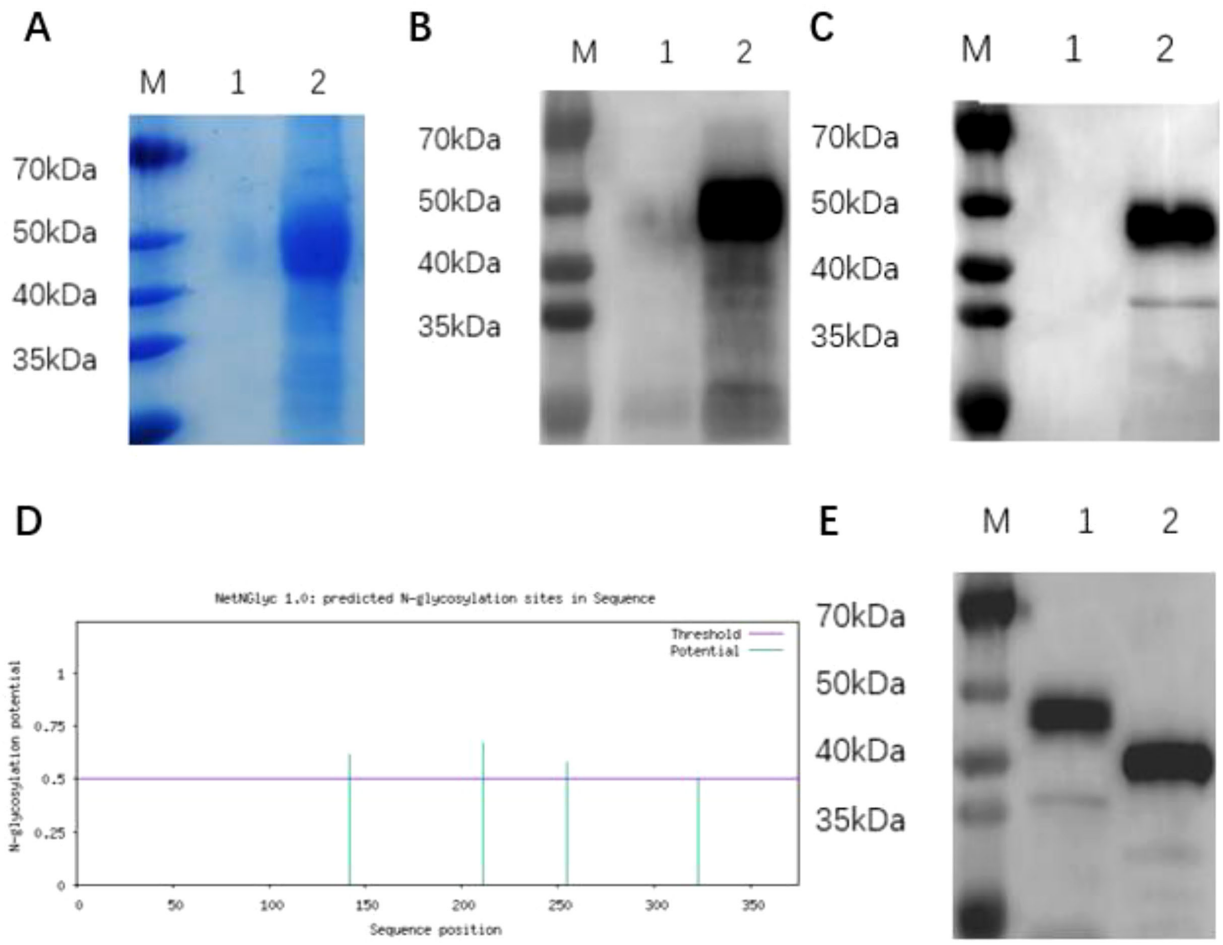
Expression and identification of BVDV rE2 protein. **(A)** SDS-PAGE analysis of recombinant BVDV rE2 protein expressed in ExpiCHO cells and stained with Coomassie Brilliant Blue. M, molecular marker; lane 1, cell supernatant containing the control plasmid pcDNA3.1; lane 2, purified BVDV rE2 protein. **(B, C)** Western blot analysis of recombinant BVDV rE2 protein using a specific monoclonal antibody against the viral E2 protein **(B)** and an anti-His monoclonal antibody **(C)**. M, molecular marker; lane 1, cell supernatant containing the control plasmid pcDNA3.1; lane 2, cell supernatant containing the plasmid pcDNA3.1-E2. **(D)** Prediction of N-linked glycosylation modification sites on the E2 protein. **(E)** SDS-PAGE analysis of deglycosylated BVDV rE2 protein. M, molecular marker; lane 1, cell supernatant containing the pcDNA3.1-E2 plasmid; lane 2, pcDNA3.1-E2 protein after deglycosylation mediated by PNGase F.

The recombinant BVDV E2 protein (fused with a His tag) expressed in ExpiCHO cells was analyzed by SDS-PAGE (**Figure 1A**) and confirmed by Western blot analysis using anti-BVDV E2 protein monoclonal antibody (**Figure 1B**) and anti-His monoclonal antibody (**Figure 1C**). The results showed that the target protein with a molecular weight of approximately 43 kDa was present in the cell supernatant but not in the negative control (**Figures 1B, C**). The target protein was purified from the cell supernatant using Ni-His affinity chromatography, yielding highly purified BVDV rE2 protein (**Figure 1A**). Analysis of the results predicted by biological software indicated that the BVDV E2 protein contains four N-linked glycosylation modification sites. Prediction by NetNGlyc 1.0 revealed potential glycosylation sites at positions 142, 211, 255, and 323. (**Figure 1D**). After deglycosylation treatment, the molecular weight of the protein changed from 43 kDa to approximately 38 kDa, indicating the presence of glycosylation sites (**Figure 1E**).

### rE2 protein−based ELISA and ROC analysis

**Figure 2:**
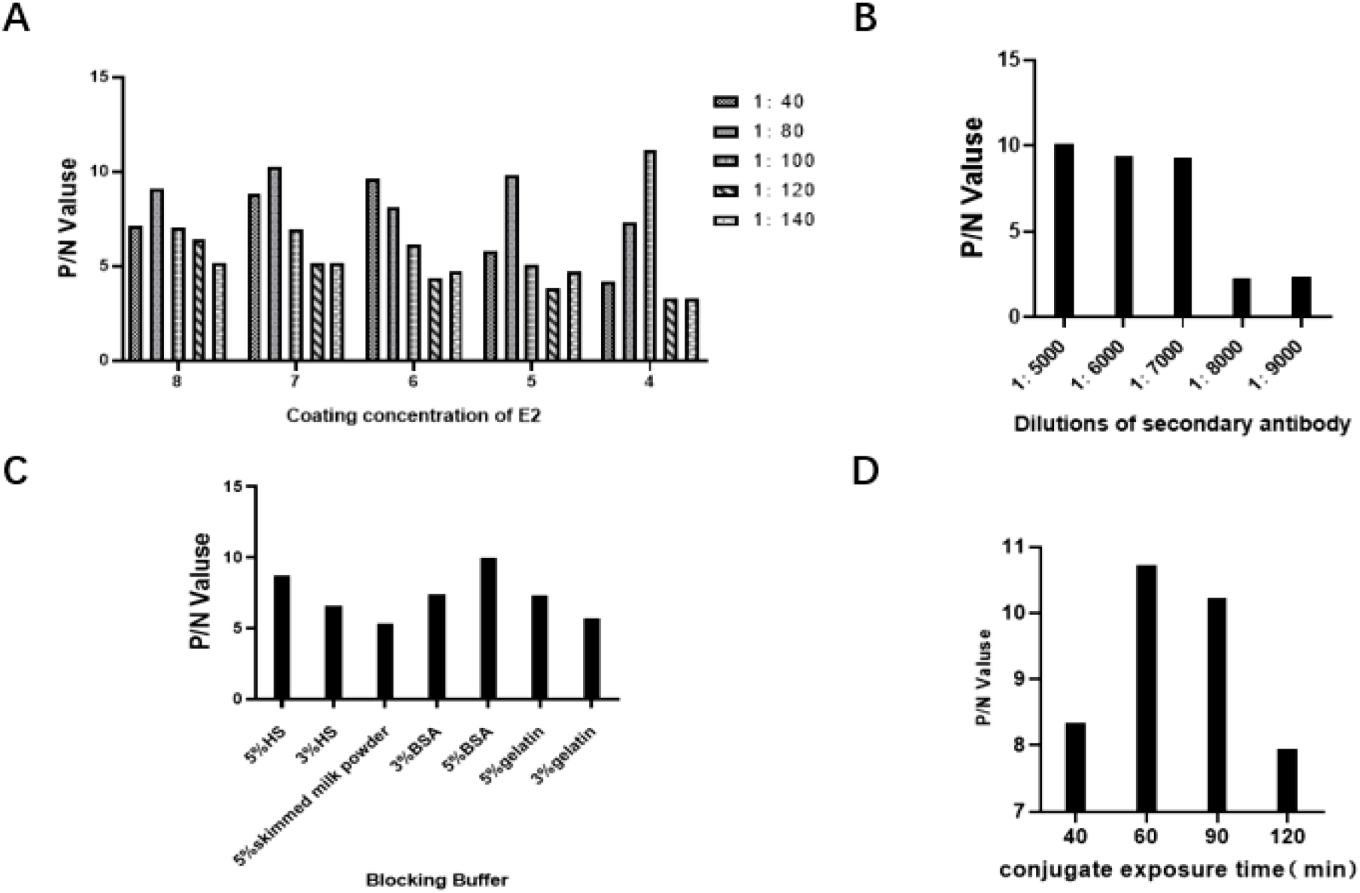
Optimization of rE2-iELISA working conditions. **(A)** Concentration of coating antigen and dilution of serum samples. **(B)** Dilution of secondary antibody. **(C)** Blocking buffers tested: 5% horse serum (5% HS), 3% horse serum (3% HS), 5% skimmed milk powder, 3% BSA, 5% BSA, 5% gelatin, and 3% gelatin. **(D)** Incubation time for secondary antibody.

The parameters of the rE2 protein-based ELISA were optimized as follows: the coating antigen concentration was set at 4 µg/mL, the serum sample dilution at 1:100(**Figure 2A**), and the secondary antibody dilution at 1:5000 (**Figure 2B**). The blocking buffer used was 5% BSA, with an incubation time of 2 hours (**Figure 2C**). This buffer effectively reduces background reactions and false positives. The optimal incubation times for secondary antibodies were determined to be 1 hour (**Figure 2D**).

**Figure 3:**
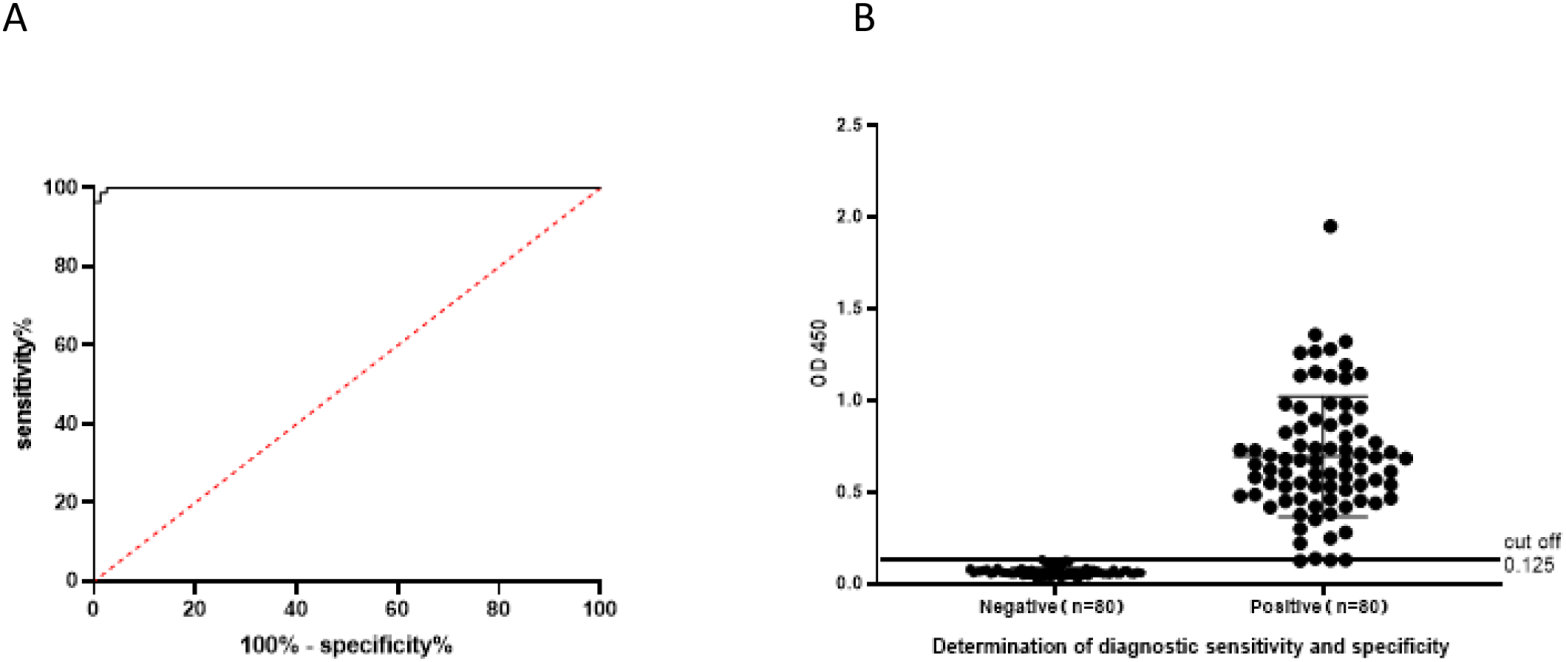
Determination of diagnostic sensitivity and specificity: **(A)** ROC curve showing the accuracy value interpreted as the area under the curve (AUC = 0.998, p < 0.001). **(B)** Cutoff value and optimal diagnostic sensitivity and specificity.

The assay involved testing 160 BVDV serum samples, including 80 negative and 80 positive cases. Using the rE2-iELISA method, a cutoff value of 0.125 was determined (**Figures 3A, B**). Samples with values ≥ 0.125 were classified as positive, while those with values < 0.125 were classified as negative. ROC curve analysis revealed an Area Under the Curve (AUC) of 0.998 (p < 0.001).

### Evaluation of specificity, sensitivity, and reproducibility of rE2-iELISA

**Figure 4:**
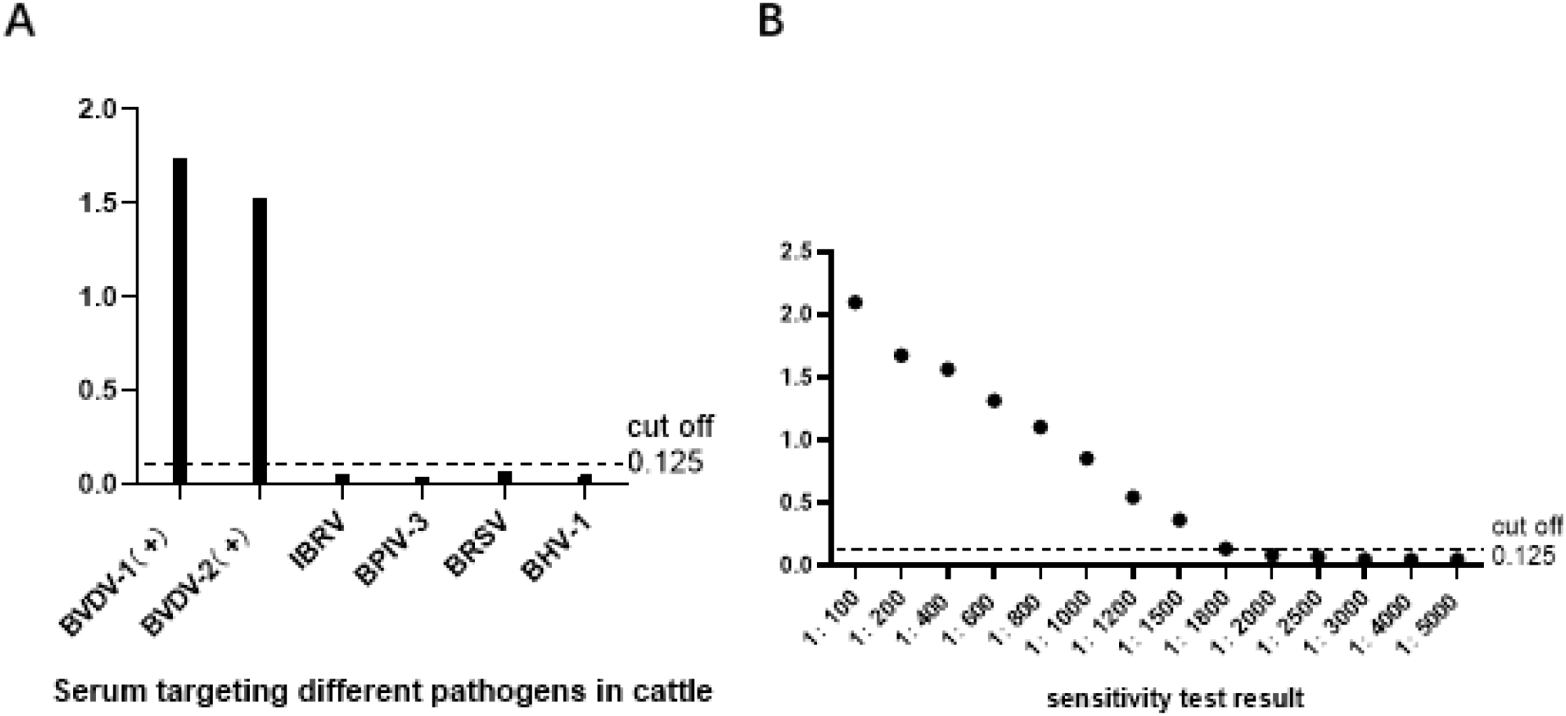
Specificity and sensitivity tests: **(A)** rE2-iELISA detected no cross-reactions with sera containing antibodies against four other pathogens. **(B)** Sensitivity test of the rE2-iELISA.

To assess the specificity of the established rE2-ELISA method, we tested serum samples for antibodies against BVDV-1, BVDV-2, IBRV, BRSV, BPIV-3, and CSFV. The results showed that only serum samples positive for BVDV-1 and BVDV-2 were detected, while all other serum samples were negative. This indicates that the rE2 ELISA method has high specificity (**Figure 4A**). Additionally, we performed a series of dilutions on BVDV-positive serum samples to assess the sensitivity of the rE2-ELISA method. The results showed that positive results could still be detected even when serum samples were diluted to 1:1500 (**Figure 4B**). Furthermore, repeatability testing of the indirect ELISA method showed an intra-batch coefficient of variation ranging from 1.38% to 3.39% and an inter-batch coefficient of variation ranging from 1.36% to 4.85%, both below 5%. This confirms that the established rE2-IELISA method is reliable and stable. (**Table 2**).

**Table 2:** Repeatability test.

Sample	Intra-batch		Inter-batch	
	SD± x¯	CV%	SD± x¯	CV%
1	0.996 ± 0.032	3.21%	0.986 ± 0.041	4.16%
2	1.321 ± 0.033	2.50%	1.325 ± 0.062	4.67%
3	0.766 ± 0.026	3.39%	0.825 ± 0.040	4.85%
4	1.021 ± 0.033	3.23%	0.996 ± 0.032	3.21%
5	1.431 ± 0.025	1.75%	1.426 ± 0.035	2.45%
6	1.16 ± 0.016	1.38%	1.13 ± 0.025	2.21%
7	1.29 ± 0.038	2.95%	1.27 ± 0.039	3.07%
8	1.07 ± 0.019	1.78%	1.32 ± 0.023	1.74%
9	1.19 ± 0.028	2.35%	0.98 ± 0.016	1.63%
10	1.26 ± 0.034	2.70%	1.77 ± 0.024	1.36%

### Validation of the rE2−iELISA

A comparative analysis of rE2-iELISA, serum neutralization test (SN), and commercial IDEXX-ELISA revealed significant differences in diagnostic performance among the three methods for detecting antibodies to bovine viral diarrhea virus (BVDV) (**Table 3**). Among 500 serum samples, the in-house rE2-iELISA demonstrated high consistency with both reference methods but exhibited minor differences in sensitivity. Compared to the SN test (430 positive and 70 negative samples), the rE2-iELISA detected 440 positive and 60 negative cases, demonstrating higher sensitivity and significant consistency (Kappa value = 0.81). WB analysis of inconsistent samples with 10 samples misclassified as false negatives by the SN test confirmed as true positives by WB. However, compared to the IDEXX-ELISA (446 positive and 54 negative samples), the rE2-iELISA showed slightly lower detection performance, with the commercial kit detecting an additional 6 positive cases confirmed by WB. The agreement between the rE2-iELISA and IDEXX-ELISA was excellent (Kappa value = 0.83), although the commercial test kit maintained a slight advantage in diagnostic accuracy. These findings indicate that the rE2-iELISA outperforms SN in terms of sensitivity and performs similarly to the IDEXX-ELISA, demonstrating its reliability while highlighting potential areas for future improvement.

**Table 3:** Comparative evaluation of BVDV antibody detection methods.

BVDV rE2−iELISA	Serum neutralization			IDEXX-ELISA		
	Positive	Negative	Total	Positive	Negative	Total
Positive	420	20	440	430	10	440
Negative	10	50	60	16	44	60
Total	430	70	500	446	54	500
Agreement	94%			94.8%		
Kappa	0.81			0.83		

## Discussion

BVDV is an important pathogen in the livestock industry, causing reproductive failure, immunosuppression, and mucosal diseases, resulting in significant economic losses (Ma et al., 2011). The virus can establish a persistent infection in calves, leading to continuous viral shedding and widespread transmission within herds (KhodakaramTafti and Farjanikish, 2017). Accurate and early detection of BVDV is crucial for disease management, as it helps prevent outbreaks and reduce the risk of viral transmission (Lee and Gillespie, 1957). Therefore, establishing reliable diagnostic methods is essential for monitoring BVDV infection and application. Currently, there are no specific drugs available for preventing or treating BVDV. Domestic and international methods primarily rely on comprehensive measures, including vaccination, pathogen and antibody detection, eliminating persistent infections in cattle, and population purification (Deng et al., 2015).

The indirect ELISA method for serum antibody detection has low requirements for personnel and equipment. It provides rapid and direct diagnostic results. This makes it highly valuable for detecting clinical samples and monitoring vaccine-induced immune antibodies, making it suitable for widespread use. As a common serological technique in biomedical research and clinical settings, ELISA can identify antigens or antibodies in the target organism through specific antigen-antibody interactions (Mahmoodi et al., 2014).

This technique can determine whether a host is infected with a pathogen or assess its immune response. The indirect ELISA method developed in this study has several advantages over traditional diagnostic methods. Its high sensitivity and specificity allow the detection of low concentrations of BVDV antibodies in serum samples. By using recombinant E2 (rE2) protein as the coating antigen, the detection specificity is enhanced by targeting key immunogenic viral proteins. The indirect ELISA method is cost-effective, easy to operate, and suitable for high-throughput screening, making it an ideal choice for large-scale monitoring projects.

Currently, most reported ELISA methods use recombinant proteins expressed in prokaryotic systems as coating antigens. The absence of post-translational modifications in prokaryotic expression systems often results in insufficient protein antigenicity. This not only complicates protein purification but may also reduce the sensitivity and specificity of ELISA detection (Elisa Walraph et al., 2018). To overcome these challenges, this study employed a eukaryotic expression system to prepare the recombinant E2 (rE2) protein of BVDV. Like mammalian cells, eukaryotic systems can perform complex post-translational modifications, such as glycosylation and phosphorylation, which are crucial for maintaining the structural and functional integrity of viral proteins like E2 (Pecora et al., 2014). In mammalian cell lines, Chinese hamster ovary (CHO) cells and human embryonic kidney 293 (HEK293) cells are most commonly used for recombinant protein production. CHO cells are favored for their high productivity, genetic stability, and ability to grow in large-scale serum-free suspension cultures (Jain et al., 2017). In this study, CHO cells were selected to express rE2, ensuring that the protein retains its native conformation and antigenic properties. As the primary immune system of BVDV, the E2 protein is critical for viral attachment and immune evasion. By producing rE2 in CHO cells, we ensured that the protein was suitable for diagnostic purposes.

The development of this indirect ELISA method lays a solid foundation for the development of commercial diagnostic kits for veterinary clinics and laboratories. Additionally, the successful expression of rE2 provides a basis for further research into the immunogenicity and protective potential of the E2 protein, which may be key to developing new BVDV vaccines. Continued optimization and validation of this method are essential to ensure its applicability across different geographical regions and BVDV genotypes.

## Conclusion

In this study, the BVDV E2 protein was expressed in CHO-S cells and purified using GE affinity chromatography on a nickel column. An indirect ELISA was developed using the rE2 protein as the diagnostic antigen to detect BVDV antibodies. Compared to the virus neutralization test (VNT), the rE2-iELISA demonstrated higher sensitivity, specificity, and consistency. Additionally, the developed method was proven to be more sensitive and cost-effective than the VNT. The rE2-iELISA holds promise as a rapid, efficient, and cost-effective tool for large-scale BVDV infection screening and control programs.

## Data Availability

The datasets presented in this study can be found in online repositories. The names of the repository/repositories and accession number(s) can be found in the article/supplementary material.
